# Targeting continuity of care and polypharmacy to reduce drug–drug interaction

**DOI:** 10.1038/s41598-020-78236-y

**Published:** 2020-12-04

**Authors:** Yi-An Weng, Chung-Yeh Deng, Christy Pu

**Affiliations:** 1 Institute of Public Health, National Yang-Ming University, 155 Li-Nong ST. Sec 2, Peitou, Taipei, Taiwan; 2 Institute of Hospital and Healthcare Administration, National Yang-Ming University, 155 Li-Nong ST. Sec 2, Peitou, Taipei, Taiwan

**Keywords:** Health care, Risk factors

## Abstract

Drug–drug interaction (DDI) is common among the elderly, and it can have detrimental effects on patients. However, how DDI can be targeted has been under-researched. This study investigates whether DDI can be reduced by targeting continuity of care (COC) through reducing polypharmacy. Population claims data of Taiwan National Health Insurance were used to conduct a 7-year-long longitudinal study on patients aged ≥ 65 years (n = 2,318,766). Mediation analysis with counterfactual method and a 4-way decomposition of the effect of COC on DDI was conducted. Mediation effect through excessive polypharmacy differed from that through lower-level polypharmacy. Compared with the low COC group, the high COC group demonstrated reduced excess relative risk of DDI by 26% (excess relative risk =  − 0.263; 95% Confidence Interval (CI) =  − 0.263 to − 0.259) to 30% (excess relative risk =  − 0.297; 95% CI =  − 0.300 to − 0.295) with excessive polypharmacy as the mediator. The risk only reduced by 8% (excess relative risk =  − 0.079; 95% CI, − 0.08 to − 0.078) to 10% (excess relative risk =  − 0.096; 95% CI, − 0.097 to − 0.095) when the mediator was changed to lower-level polypharmacy. The effect of COC on DDI was mediated by polypharmacy, and the mediation effect was higher with excessive polypharmacy. Therefore, to reduce DDI in the elderly population, different policy interventions should be designed by considering polypharmacy levels to maximize the positive effect of COC on DDI.

## Introduction

Drug–drug interaction (DDI) events are common in the elderly population. The prevalence of clinically relevant DDI may be as high as 28.1% in the elderly population^1^. However, DDI-related adverse reactions are generally predictable and amendable^2^. Many avoidable hospital admissions for drug-related toxicity were due to preventable DDIs^3^.

Continuity of care (COC) comprises 2 elements: how patients experience integration of services and how care is delivered over time^4^. In the recent years, enhancing patient COC has become one of the central themes in patient care. Greater COC is associated with fewer emergency department visits and hospital admissions and lower health care costs, which can be partially explained by the reduction of inappropriate medication use^5^. Higher COC is also related to better medication adherence and a reduction in drug-related problems such as inadequate dosing and inappropriate regimens^6,7^.

High COC can reduce DDI event frequency, and this effect is greater in population with multiple comorbidities^8^. Despite its medical benefits, research on the mechanism through which high COC acts as a protective factor against DDI incidences remains insufficient. Knowledge on the mechanism of how COC affects DDI is essential for 2 reasons. First, ignoring the mediation effect can likely lead to biased estimates and render study results invalid. Second, mediation analysis allows policies to maximize the effects of COC on DDIs through the intervention of a mediator and hence improve an intervention’s components. Such studies on COC-DDI relationship are thus scant.

Polypharmacy, which refers to the simultaneous usage of multiple medications, has been found to be a risk factor for DDI in older people^1,2^. DDI events can occur even when all the prescription guidelines are followed^9^. With increasing number of comorbidities and physicians involved, polypharmacy has become an inevitable consequence for patients^10^. The greater prevalence of multi-comorbidities in older people makes polypharmacy a common phenomenon for this population^9^. In an outpatient setting, 39.4% of the elderly population were exposed to polypharmacy^11^. Managing polypharmacy has been an issue in patient care in the elderly population.

Increasing COC reduces the number of medications. A retrospective study demonstrated that both physician continuity and site continuity exert protective effect over medication duplication, and the effect was greater for elderly patients with multi-comorbidities^12^. A Japanese study discovered that an older patient without a regular physician was 2.5 times more likely to experience polypharmacy than those who had one^13^.

The association between greater COC and positive patient outcomes has been well studied and investigated in many settings. However, the mechanism through which COC affects patient outcomes and reduces DDI has not been investigated. Polypharmacy is a DDI risk factor that is not only influential but also identifiable and amendable. Identifying its role in patient care is essential for elderly patients. The relationship between COC, polypharmacy, and DDI must be investigated to improve patient outcomes for elderly people. The results of our study thus possess policy relevance for the improvement of elderly patients’ outcomes. In addition to possessing policy relevance, this study addresses a shortcoming of other research (i.e., failure to use rigorous statistical methods for estimating mediation effects). This study tested whether the effect of COC on DDI is mediated by polypharmacy, and if so, what is the magnitude of the mediating effect.

## Methods

In this study, we used the Taiwan National Health Insurance (NHI) claims database, managed by Taiwan’s Ministry of Health. The NHI program has been implemented in Taiwan since 1995 and is compulsory for all citizens. To date, the enrollment rate has reached 99.6% of the population. All the medical records from both inpatient and outpatient settings, including all prescriptions and procedures, are included in the database. The NHI claims data have been used frequently for studies involving drugs and pharmaceuticals because it provides accurate information on prescriptions, which is generally absent in the survey data generally^5,6,14^. This study was approved by the Institutional Review Board of National Yang-Ming University.

### Inclusion criteria

Here, the elderly population was defined as people who are ≥ 65 years, and all records from the eligible patients’ outpatient visits were extracted. Patients who met the following inclusion criteria were enrolled: (1) > 1-year-long survival since 2011 and (2) ≥ 3 physician visits in the same year during the study period. These criteria were necessary as the COC index (COCI) can only be calculated within a time interval and with a reasonable number of visits^15^. By using these inclusion criteria, a total of 2,318,766 people were included in 2011. This cohort was then studied for 7 years until 2017.

### Exposure: COC

We used the COCI to measure care continuity. This measurement is more suitable for health systems with a high number of outpatient visits^16^. COCI measures the concentration of visits in a certain period^17^. Physician COCI is thus calculated as follows^18^:

$$\mathrm{Physician\,\, COCI}=\frac{{\sum }_{i=1}^{p}{n}_{i}^{2}-N}{N(N-1)},$$

where *n*_*i*_ is the number of visits to the given physician *i*, *p* the number of the physicians that the patient visited, and *N* the total number of physician visits.

### Mediator and outcome variables

To analyze polypharmacy and DDI, only Western medicine and drugs were included. Drugs that required only topical application, Chinese herbal medications, and over-the-counter medications were excluded. To identify the medication prescribed for each patient, we applied the Anatomical Therapeutic Chemical code as the classification index.

Studies have used various cutoff points for the number of medications to define polypharmacy^19^. However, most studies have used 5 medications as the cutoff points^11,20,21^; hence, we also employed this cutoff for our study to ensure comparability. Thus, patients who were simultaneously on ≥ 5 medication types in a 3-month window were considered to be having polypharmacy; those who were being administered ≥ 10 medication types were defined as having excessive polypharmacy^14,22^. DDIs can be examined using different approaches, such as by using knowledge bases (KBs)^2,23^ and using the system used for clinical evaluation in a single country^24,25^. To account for the clinical severity of DDIs, professionals were recruited for evaluating the harm to the human body^26,27^.

This study used a list of 15 types of DDIs identified as clinically significant by Phansalkar et al.^28^; this list has been adopted by several studies and in practice^8,29,30^. We selected this list as our target DDIs for the following reasons: (1) the information on the DDIs was cross-compared with the different KBs, (2) the list was strictly reviewed by a panel of experts from different backgrounds who had reached the consensus that the patients were critical and should be integrated into the clinical decision support system, and (3) the list presented the drug-class level of the DDI pairs, which allowed us to link it with the information we had in our data set. Some modifications were made to the list to exclude those combinations that were not covered by insurance or were not applied to the population. Only 10 types of DDIs were consequently included in the subsequent analysis. A DDI event was defined as 2 target drugs of a DDI pairs that were prescribed, with treatment periods that overlapped for at least 1 day.

### Statistical analysis

To test the hypothesis that polypharmacy acts as a mediator between COC and DDI, we applied a counterfactual method because the exposure and mediator were measured simultaneously^31^. The product method and the difference method are the 2 traditional methods for mediation analysis. The difference method entails a comparison between the original effect of the exposure on the outcome and the one adjusted for the mediator, and the product method estimates the mediation effect by multiplying the effect of the exposure on the outcome and the effect of the mediator on the outcome after adjustments for the exposure and other covariates^32,33^. The traditional methods have been criticized for their lack of causal interpretation that leads to a biased estimation of the indirect effect with incorrect conclusion for mediation when applying a logistic regression in nonrare outcomes or with an exposure-mediator interaction term^34,35^.

We used counterfactual method with 4-way decomposition for the mediation analysis^36^, which is an advanced method that decomposes the overall effect into 4 components: controlled direct effect (CDE), reference interaction (INTref), mediated interaction (INTmed), and pure indirect effect (PIE). Two regression models are required for the estimation: (1) the outcome model, a function of the exposure, mediator, and interaction between theses and the confounders, and (2) the mediator model, a function of the exposure and confounders^37^. Logistic regressions were estimated for both models. We defined high and low COC groups on the basis of the median of the COCI. DDI events and polypharmacy status were treated as dichotomous variables (with or without DDIs or polypharmacy).

For sensitivity analysis, we redefined COC, polypharmacy, and DDI to determine whether our results were robust to different specifications. For COC, we recalculated the COCI using 2 visits, consistent with a previous study^38^. For defining polypharmacy and excessive polypharmacy, we used a longer time window, such that if a patient had received ≥ 5 medications repeatedly and persistently for > 181 days, it would be defined as persistent polypharmacy^39^. This alternative time window was also applied to define persistent excessive polypharmacy^22^. DDI was defined as the presence of a combination for ≥ 2 days (rather than 1 day) to ensure that our results were robust because of the stricter definition. We also regrouped COC by using tertiles to create low, intermediate, and high COC groups, which was consistent with previous studies^5,40^.

For subgroup analysis (results not shown), we selected the 2 demographic characteristics that had been confirmed to have impact on COC and polypharmacy. A Korean retrospective study that focused on patients aged ≥ 65 years with diabetes, hypertension, asthma, and chronic obstructive pulmonary disorder found that COC was higher in men than in women and tended to decrease with age^40^. The differences between men and women in terms of the attitude toward medications and doctor-seeking behaviors were significant. Studies have revealed that women have higher physicians visit rates and have more positive attitudes toward medication therapies than men do^41,42^, and older people are more likely to receive polypharmaceutical therapies than younger people do^42^.

All statistical analyses were conducted using STATA MP Environment (StataCorp. 2017. *Stata Statistical Software: Release 15.* College Station, TX: StataCorp LLC.).

## Results

Table 1 lists the characteristics of the study population at baseline categorized by low and high COC groups. Significant differences were observed for sex and age between the 2 groups (*P* < .001). The number of physician visits and prescribed medications and the proportion of patients with polypharmacy and DDI events were significantly lower in the high COC group than in the low COC group (*P* < .001). During the study period, there was a little fluctuation in the means of the COCI. The changes of the proportions of polypharmacy cases and excessive polypharmacy cases were little. However, the percentage of DDI events in the study population increased over the years.

**Table 1:** Baseline patient characteristics.

Variables	Low COC group	High COC group	*P* value^a^	All patients
N	1,159,069	1,159,697		2,318,766
COCI [mean(SD)]	0.15(0.06)	0.46(0.21)	< 0.001	0.30(0.22)
Age in 2011 [mean(SD)]	74.71(6.94)	74.72(6.96)	0.382	74.71(6.95)
**Age group [n (%)]**			< 0.001	
65–74	629,334(54.30)	634,364(54.70)		1,263,698(54.50)
75–84	412,402(35.58)	406,122(35.02)		818,524(35.30)
85+	117,333(10.12)	119,211(10.28)		236,544(10.20)
**Gender [n (%)]**			< 0.001	
Male	532,303(49.26)	548,359(50.74)		1,080,662(46.61)
Female	626,766(50.62)	611,338(49.38)		1,238,104(53.39)
Physician visits [mean(SD)]	35.05(23.44)	25.25(17.91)	< 0.001	30.15(21.42)
**Physician visits frequency [n(%)]**			< 0.001	
Low	306,827(26.47)	487,507(42.04)		794,334(34.26)
Intermediate	347,785(30.01)	422,063(36.39)		769,848(33.20)
High	504,457(43.52)	250,127(21.57)		754,584(32.54)
No. of prescribed medications [mean(SD)]	11.29(6.41)	8.17(4.81)	< 0.001	9.73(5.88)
**Polypharmacy status [n(%)]**			< 0.001	
No	66,193(5.71)	141,473(12.20)		207,666(8.96)
Yes	1,092,876(94.29)	1,018,224(87.80)		2,111,100(91.04)
**Drug–drug interaction events [n(%)]**			< 0.001	
No	1,104,885(95.32)	1,135,212(97.88)		2,240,097(96.60)
Yes	54,184(4.67)	24,485(2.11)		78,669(3.39)

*COCI* continuity of care index, *SD* standard deviation.

^a^Chi-square tests were used for comparisons between age group, gender, physician visits frequency, polypharmacy status, and DDI events, and *t* tests were used to compare COCI, physician visits, and number of prescribed medications.

Tables 2 and 3 present the result of the 4-way decomposition for the relationship between COC, polypharmacy or excessive polypharmacy, and DDI. These estimates represent the excess relative risk of DDI due to the total effect, CDE, INTref, INTmed and PIE, signifying a change in the risk percentage compared with the reference group. The CDE was estimated at a fixed level of the mediator. In Table 3, polypharmacy was treated as the mediator, and the total excess relative risk was estimated to be − 0.565 (*P* < .001) in 2011, indicating that the cases of DDI events decreased by 56.5% in the high COC group than in the low COC group. Similar results were found when we used excessive polypharmacy as the mediator; the total excess relative risk was − 0.570 (*P* < .001; Table 3). The excess relative risk of COC on DDI events due to the PIE (pure mediation) of polypharmacy was significant: the one due to polypharmacy ranged from − 0.079 to − 0.096, and the one due to excessive polypharmacy was greater, ranging from − 0.261 to − 0.297 (both *P* < .001). The excess relative risk is attributable to reference interaction (interaction only) and mediated interaction (mediation and interaction); however, both of them slightly increased the excessive risk of COC on DDI events.

**Table 2:** Four-way decomposition of the relationship between COC, polypharmacy, and DDI.

	2011		2012		2013		2014		2015		2016		2017	
	β^a,b^	*p*	β	*p*	β	*p*	β	*p*	β	*p*	β	*p*	β	*p*
**Mediator at 0**														
Total excess RR	− 0.565	< 0.001	− 0.576	< 0.001	− 0.581	< 0.001	− 0.602	< 0.001	− 0.619	< 0.001	− 0.612	< 0.001	− 0.622	< 0.001
Excess RR due to controlled direct effect	0.004	0.193	0.004	0.131	0.008	0.003	0.001	0.878	0.007	0.020	0.003	0.251	0.005	0.112
Excess RR due to reference interaction	− 0.533	< 0.001	− 0.546	< 0.001	− 0.551	< 0.001	− 0.564	< 0.001	− 0.589	< 0.001	− 0.578	< 0.001	− 0.589	< 0.001
Excess RR due to mediated interaction	0.043	< 0.001	0.043	< 0.001	0.048	< 0.001	0.052	< 0.001	0.055	< 0.001	0.053	< 0.001	0.057	< 0.001
Excess RR due to pure indirect effect	− 0.079	< 0.001	− 0.078	< 0.001	− 0.086	< 0.001	− 0.090	< 0.001	− 0.092	< 0.001	− 0.091	< 0.001	− 0.096	< 0.001
**Mediator at 1**														
Total excess RR	− 0.565	< 0.001	− 0.576	< 0.001	− 0.581	< 0.001	− 0.602	< 0.001	− 0.619	< 0.001	− 0.612	< 0.001	− 0.622	< 0.001
Excess RR due to controlled direct effect	− 0.568	< 0.001	− 0.579	< 0.001	− 0.583	< 0.001	− 0.602	< 0.001	− 0.621	< 0.001	− 0.612	< 0.001	− 0.620	< 0.001
Excess RR due to reference interaction	0.039	< 0.001	0.038	< 0.001	0.040	< 0.001	0.039	< 0.001	0.039	< 0.001	0.036	< 0.001	0.036	< 0.001
Excess RR due to mediated interaction	0.043	< 0.001	0.043	< 0.001	0.048	< 0.001	0.052	< 0.001	0.055	< 0.001	0.053	< 0.001	0.057	< 0.001
Excess RR due to pure indirect effect	− 0.079	< 0.001	− 0.078	< 0.001	− 0.086	< 0.001	− 0.090	< 0.001	− 0.092	< 0.001	− 0.091	< 0.001	− 0.096	< 0.001

*RR* relative risk, *COC* continuity of care, *DDI* drug–drug interaction.

^a^β: model estimates.

^b^Low COC group is the reference group.

**Table 3:** Four-way decomposition of relationship between COC, excessive polypharmacy, and DDI.

	2011		2012		2013		2014		2015		2016		2017	
	β^a,b^	*p*	β	*p*	β	*p*	β	*p*	β	*p*	β	*p*	β	*p*
**Mediator at 0**														
Total excess RR	− 0.570	< 0.001	− 0.581	< 0.001	− 0.586	< 0.001	− 0.606	< 0.001	− 0.623	< 0.001	− 0.616	< 0.001	− 0.625	< 0.001
Excess RR due to controlled direct effect	0.001	0.767	− 0.002	0.635	− 0.001	0.794	− 0.011	0.002	− 0.010	0.004	− 0.015	< 0.001	− 0.019	< 0.001
Excess RR due to reference interaction	− 0.437	< 0.001	− 0.449	< 0.001	− 0.444	< 0.001	− 0.453	< 0.001	− 0.475	< 0.001	− 0.462	< 0.001	− 0.466	< 0.001
Excess RR due to mediated interaction	0.127	< 0.001	0.130	< 0.001	0.139	< 0.001	0.145	< 0.001	0.156	< 0.001	0.151	< 0.001	0.157	< 0.001
Excess RR due to pure indirect effect	− 0.261	< 0.001	− 0.261	< 0.001	− 0.280	< 0.001	− 0.287	< 0.001	− 0.294	< 0.001	− 0.290	< 0.001	− 0.297	< 0.001
**Mediator at 1**														
Total excess RR	− 0.570	< 0.001	− 0.581	< 0.001	− 0.586	< 0.001	− 0.606	< 0.001	− 0.623	< 0.001	− 0.616	< 0.001	− 0.625	< 0.001
Excess RR due to controlled direct effect	− 0.590	< 0.001	− 0.600	< 0.001	− 0.600	< 0.001	− 0.615	< 0.001	− 0.639	< 0.001	− 0.620	< 0.001	− 0.625	< 0.001
Excess RR due to reference interaction	0.154	< 0.001	0.150	< 0.001	0.155	< 0.001	0.152	< 0.001	0.154	< 0.001	0.144	< 0.001	0.140	< 0.001
Excess RR due to mediated interaction	0.127	< 0.001	0.130	< 0.001	0.139	< 0.001	0.145	< 0.001	0.156	< 0.001	0.151	< 0.001	0.157	< 0.001
Excess RR due to pure indirect effect	− 0.261	< 0.001	− 0.261	< 0.001	− 0.280	< 0.001	− 0.287	< 0.001	− 0.294	< 0.001	− 0.290	< 0.001	− 0.297	< 0.001

*RR* relative risk, *COC* continuity of care, *DDI* drug–drug interaction.

^a^β: model estimates.

^b^Low COC group is the reference group.

**Figure 1:**
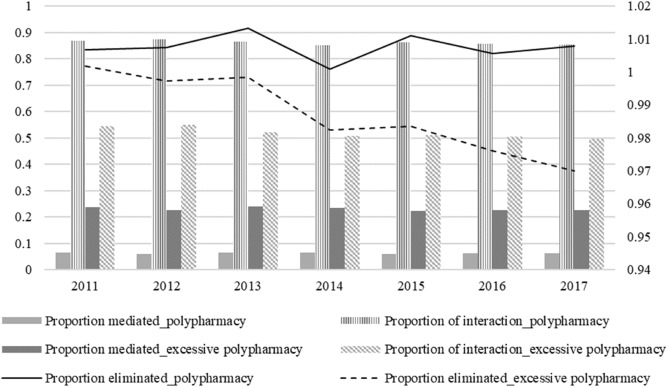
Policy-relevant proportions using different mediators. This figure demonstrated three policy-relevant proportions of the effect of COC on the DDI events: the proportion eliminated (INTref + INTmed + PIE/TE), the proportion mediated (INTmed + PIE/TE) and the proportion of interaction (INTref + INTmed/TE). With different mediators, the constitute of the effect of COC on DDI events differs. The proportion mediated was higher in the model with excessive polypharmacy (≥ 10 medications concurrently used) than the one with polypharmacy (≥ 5 medications concurrently used); while the proportion of interaction was greater treating polypharmacy as mediator instead. The proportion eliminated declined during the study period using excessive polypharmacy as a mediator.

Figure 1 presents the policy-relevant proportions, setting the mediator at neither polypharmacy nor excessive polypharmacy. The proportion mediated demonstrated the importance of the mediation pathway to explain the exposure’s effect on the outcome^43^. The proportion eliminated captured the portion of the exposure’s effect on the outcome that would be eliminated through a policy that intervened and fixed the mediator to a certain level^43^. The proportion of interaction is the sum of reference interaction and mediated interaction, which showed how exposure and mediator affect the outcome variable together. We found that the proportion mediated was greater when excessive polypharmacy was used as a mediator than when polypharmacy was used. Not much change was observed in the proportion mediated during the study period. The proportion eliminated indicated that in the absence of polypharmacy, there would be no direct protection from COC, which can be harmful. However, controlling excessive polypharmacy can be benefited through enhanced COC. The proportion of interaction was greater using polypharmacy as mediator, suggesting that the interaction between COC and polypharmacy played a more essential part in preventing DDI than excessive polypharmacy.

We stratified our analysis by sex and age and compared how the effect differed among the groups. In the analysis for sex, we found that in both models, the proportion of interaction effect was higher in women than in men, while the proportion mediated showed an opposite effect.

To analyze the effect by age, we divided the study population into 3 groups: those aged 65 to 74, 75 to 84, and ≥ 85 years. We found a difference in the proportion mediated for different mediators and age groups. The proportion mediated was the highest in the group aged ≥ 85 years using excessive polypharmacy as the mediator, and it was the highest in the group aged 65 to 74 years using polypharmacy (instead of excessive polypharmacy) as the mediator. The differences between the proportions of interaction were nonsignificant in different age groups.

### Sensitivity analysis

To test the robustness of our result, we conducted a sensitivity analysis by altering the definition of the exposure and the outcome, as described in the Methods section. Our results remained robust for all these tests, that is, higher COC was related to less DDI events, and the relationship was partially mediated by polypharmacy (tables for subgroup and sensitivity analyses are not shown; results are available upon request).

We redefined polypharmacy and excessive polypharmacy as the persistent status that lasted for the entire year, and the results of the proportion eliminated and proportion mediated were different. The proportion mediated was much lower than that suggested by the original definition in both polypharmacy and excessive polypharmacy. The proportion mediated was lower in persistent excessive polypharmacy than in excessive polypharmacy. However, there was only a slight difference between persistent polypharmacy and polypharmacy.

## Discussion

In this study, we investigated whether polypharmacy mediates the effect of COC on DDI in older patients. We found significant partial mediation effect of polypharmacy, and this mediation effect was higher when excessive polypharmacy was defined as the mediator. Our study provides evidence for maximizing the effect of COC on reducing DDI by intervening on polypharmacy. Through prescription of appropriate medication, inappropriate polypharmacy can be avoided^44^.

Our results suggest that multiple drug use is common in the elderly population, and this problem is worse in Taiwan than in other countries and regions^11,45,46^. The high prevalence of polypharmacy in Taiwan is potentially attributable to high physician visit frequency. Our results demonstrated that the average number of physician visits was 30 and that approximately two-thirds of the study population had > 17 physician visits in 1 year. Because of the lack of a proper referral system in Taiwan, patients may self-refer to physicians, leading to low COC^5^. We found a negative association between COC and polypharmacy; that is, high COC prevented polypharmacy in patients. In addition, polypharmacy and DDI were positively correlated, confirming that polypharmacy is a risk factor for DDI.

In the mediation analysis, we found that enhancing COC has protective effects for DDI events. However, when decomposing the total effect, polypharmacy and excessive polypharmacy exerted effects of different magnitudes. One study that analyzed patients with hypertension among Medicare beneficiaries found that greater COC causes elderly people to purchase more drugs for hypertension treatment^47^. This provides evidence for the possible existence of a mediation effect of polypharmacy. In our study, when polypharmacy and excessive polypharmacy were eliminated, the effect of COC on DDI events occurred primarily because of the interaction between COC and polypharmacy status. Although polypharmacy or excessive polypharmacy was presented, the protective effect could be attributed to the direct effect of COC. Furthermore, the level of interaction and mediation effect between polypharmacy and excessive polypharmacy was different. The proportion of pure mediation through excessive polypharmacy was higher than that through polypharmacy. The interaction between COC and excessive polypharmacy was greater than that between COC and polypharmacy. Thus, identifying the polypharmacy status of a patient may be essential. For patients having polypharmacy, enhancing their COC could be sufficient because direct effect constitutes a large portion of the effect. However, for excessive polypharmacy patients, enhancing their COC might not be enough. If physicians are unaware of the possible DDI incidence, they might prescribe more medications for symptom control, and this explains the positive effect of the COC-excessive polypharmacy interaction on DDI. Measures other than enhancing COC must be considered to manage the DDI of patients with excessive polypharmacy.

In the subgroup analysis, we selected sex and age as the criteria for grouping because members of different sex have been found to demonstrate different health care-seeking behaviors^40–42^. Our study revealed that the components of COC’s effect on DDI events were different for men and women. Mediation is the effect whereby exposure affects the outcome through the mediator; interaction describes how the effect of the exposure on the outcome is modified by the mediator. Women consult physicians more than men do^41^, which is a potential explanation for our baseline description of lower COC among women than men. Other studies have revealed that women are more likely to use multiple drugs than men^48–50^; and a more positive attitude toward medications in women may lead to greater willingness to use drugs^42^. As a result, the enhancement of COC might exert a greater effect on medication management among women, further reducing the frequency of DDI events in women. In the analysis of the effect of COC on DDI events for different age groups, the effect components had a higher difference in the models that used excessive polypharmacy as the mediator. The proportion eliminated of the group aged ≥ 85 years was lower than that of other age groups. Assumption for the change could thus be made that for this age group, the treatment might be more conservative and regular^14^. Hence, visiting a regular physician familiar with the patient in addition to the drug history might be helpful.

Managing the number of medications could be an effective way to enhance the positive effect of COC on reducing DDI. Recent studies and policies have primarily emphasized on managing potential inappropriate medications. Many guidelines such as Beer’s criteria and STOPP/START criteria were developed to reduce the number of medications administered by reviewing the medications and evaluating the appropriateness^51,52^. Nevertheless, appropriate medications might impose other risks on patients, such as DDIs. To manage this problem, other inventions dealing with DDI-related polypharmacy should be considered. Studies have shown that pharmacists may be crucial in reducing the polypharmacy that imposes risk on patients^27,53^. Regular reconciliation of medications can reduce the number of drugs and lower the burden of polypharmacy. Establishing monitoring plans for DDIs can ensure patient safety.

Nowadays, electronic systems have been widely adopted in medical facilities. NHI PharmaCloud system, established in 2013, served as an effective tool for medication evaluation because it is connected to the NHI claim system, which allows for the sharing of prescription information^54^. Despite the platform’s ability to improve the health care quality, a survey conducted among physicians indicated resistance to use the system^55^. Improving the information quality also enhances people’s intention to use the tool. Setting up reminders for reviewing polypharmacy status may be helpful in alerting the medical staff to evaluate the medication usage and increase the awareness of potential DDIs.

This study had limitations that should be considered when interpreting the results. First, we only used those patient characteristics (eg, sex and age) that were available in the claim database for the covariates. Other patient characteristics (eg, education) that may alter their physician-seeking and medication-using behaviors were not adjusted in this study^46^. Second, the claims data provided no information on medication adherence and only allowed us to identify the prescribed DDI combinations. However, given that some DDIs can be fatal, administering these combinations of DDI drugs on patients should be a public health concern, regardless of the levels of medication adherence. Finally, our results may not be generalized to health care systems that mainly adopt private insurances or sound referral systems. Different health care systems can produce different COC with varying COC effects. Our results provided useful information for countries with NHI^38^. We only involved some variables that had been well-studied in the COC study. However, other factors can influence the effect of COC on DDI. However, we believed that the effect of these factors are minimal.

Both DDI and polypharmacy are common phenomena for the elderly population, and the magnitude of polypharmacy influences the quality of care. In this study, we found that polypharmacy was a partial mediator in the relationship between COC and DDI, and the effect of mediation differed with different magnitudes of polypharmacy. This provided the empirical proof supporting the claim that polypharmacy should be addressed in patient-caring^53^. Moreover, different interventions should be developed categorized by age, gender, and polypharmacy status.
